# The inevitable! An emergence of a new influenza virus

**DOI:** 10.4103/1817-1737.53344

**Published:** 2009

**Authors:** Hanan H. Balkhy

**Affiliations:** *Department of Pediatrics and Pediatric Infectious Diseases, King Abdullah International Medical Research Center, King Saud bin Abdulaziz University for Health Science, Saudi Arabia*

A public health emergency, due to a novel influenza virus that affects humans, was declared by the WHO on April 25, 2009. On June 11, 2009, Dr. Margaret Chan, Director-General of the World Health Organization, raised the pandemic alert level to level 6 after the identification of swine-origin influenza virus (S-OIV) cases in more than one WHO region.[1] This is the first global pandemic of the twenty-first century.

What will happen in the next months can be predicted from previous pandemics and their progress. Miller et al., elegantly describe signature features of four major pandemics of the nineteenth and twentieth centuries: the 1889 A/H1N, the 1918 A/H2N2, the 1957 A/H3N2, and the 1968 pandemics.[2] The signature feature, which defines a pandemic, is a shift in the virus subtype. Other features include: a shift in the highest mortality rates to the younger population; higher human to human transmission rates in comparison to seasonal influenza; the occurrence of successive pandemic waves, where early milder outbreaks allow for partial immunization of the population, and finally variations in the geographical impact of the pandemic, which may be attributed to the presence of partially immunized individuals during the pandemic early waves, climate differences, and social variations; such as mixing and crowding.[2]

To date, there are more than 50,000 confirmed cases in more than 90 countries around the globe, with more than 230 deaths. In the kingdom of Saudi Arabia, 39 cases have been diagnosed, but no S-OIV-related deaths have occurred. Overall, cases continue to be mild, and death rates continue to be low.[1]

## The Hajj Season and An Emerging Virus

The challenges faced by policy makers are decisions regarding vaccine production as well as vaccine and medication purchases and distribution, all of which are critical and difficult. Many of these decisions must be based on virus evolution and disease severity, but this information is not fully available.[3] Furthermore, the rationale behind resource allocation is another challenge. Both influenza and pandemic viruses evolve between seasons. With the variation in the influenza seasonality of the Northern and Southern hemisphere, it is difficult to predict how the virus will evolve in the coming months. Sero-epidemiological surveys may help estimate the extent of disease transmission and severity; however, such studies may be limited to wealthy countries.[3]

The Kingdom of Saudi Arabia has made additional preparations this year for the upcoming Hajj and Omra seasons by creating scientific committees under the direction of the Ministry of Health, to decide on proper precautionary measures to ensure a safe pilgrimage. These decisions are to be based on the present and evolving scientific evidence on the pandemic as it becomes available.[4]

## Clinical Presentation and Prevention

Disease transmission can potentially occur through direct droplet nuclei or surface contamination with the virus from respiratory secretions. There is also the potential of feco-oral transmission, as fecal shedding is expected in patients with diarrhea.

The full clinical spectrum and natural history of the disease is not yet determined. Most cases have thus far been mild and limited in severity. Patients at risk for severe and fatal disease are predicted to be those who are at risk for severe seasonal influenza. Of the confirmed cases of S-OIV reported in the USA, most presented as mild, uncomplicated, febrile respiratory illnesses.[5] Among the infected patients, 40% were in the age range of 10 – 18 years, and the median age was 20 years. Fever and cough were present in over 90% of the cases, while vomiting and diarrhea were present only in 25%. There was a history of recent travel to Mexico within seven days of onset of the illness in 18% (68 / 381) of the cases. As of June 22, the mortality rate among cases from around the globe has been variable.[1] Many countries had no reported deaths, while in the USA, 87 (0.41%) deaths were reported among 21,449 confirmed cases. The highest number of deaths, 113 out of 7,624 confirmed cases (1.5%), were reported in Mexico.

Healthcare providers should obtain a nasopharyngeal swab from suspected cases of swine influenza, for testing. Protocols for specimen collection and transportation should be followed according to the guidelines of the local health department. In the Kingdom of Saudi Arabia, the local guidelines are provided by the Ministry of Health; these guidelines have been published and distributed to all health care facilities and are available on their website.[4] Management of patients with confirmed S-OIV includes symptomatic therapy and the initiation of neuraminidase inhibitors. Additionally, the website of the Center for Disease Control and Prevention (CDC) continuously updates management options for governments to adopt.[6] Modes of prevention are particularly needed in crowded areas, such as airports, where the use of a facemask and strict hygiene at all times are the primary methods of prevention.[7]

## Conclusion

There is now a clear identification of a pandemic due to a novel swine H1N1 influenza virus; this pandemic includes cases from the Kingdom of Saudi Arabia. The high number of cases in specific geographical regions may be explained by the focused epidemiological survey, where more cases could be identified if an enhanced global surveillance took place. For our local concern in the Kingdom, it is imperative that global surveillance continues and that poor countries, from which pilgrims originate, are included in such a surveillance. Such information would assist the Kingdom and the Muslim world in making informed decisions on the annual pilgrimage, for instance, vaccine prerequisites for obtaining a Hajj visa or the exclusion of certain countries from participating in the annual Hajj; which is an extreme measure that has not been taken yet. Finally, continuous surveillance of humans and animals affected by influenza viruses within the Kingdom has become a necessity and not a luxury.
